# An Intestinal Melanoma of Unknown Origin Presenting as an Intussusception: A Case Report of a Rare Entity

**DOI:** 10.7759/cureus.80032

**Published:** 2025-03-04

**Authors:** Diego Ontiveros Ramírez, Carlos Alberto Ramírez Alvarado, Mario Alberto Ramírez Gonzalez, Fernando Carballar Mejía, Sergio Maldonado Mares

**Affiliations:** 1 Surgical Gastroenterology, Hospital Regional Issste, Leon, MEX; 2 Oncologic Surgery, Hospital Angeles León, Leon, MEX

**Keywords:** gastrointestinal melanoma, intestinal intussusception, melanoma, melanoma of unknown primary, metastasic melanoma

## Abstract

Melanoma involving the gastrointestinal (GI) tract is an exceedingly rare and clinically challenging entity, often presenting with nonspecific symptoms that can delay diagnosis and treatment. This report describes the case of a 74-year-old male with a history of melanoma of unknown primary origin who presented with acute intestinal obstruction secondary to metastatic melanoma in the small intestine. The patient’s clinical course was complicated by intermittent subocclusion, anemia, and hemodynamic instability, culminating in exploratory laparotomy and resection of the affected intestinal segment. Histopathological examination confirmed metastatic melanoma, positive for SOX10, HMB45, and Ki67 markers. This case highlights the diagnostic and therapeutic challenges of GI melanoma, emphasizing the importance of considering rare malignancies in patients with atypical abdominal symptoms, particularly those with a history of melanoma. The discussion underscores the aggressive nature of GI melanoma, the role of surgery as the primary treatment modality, and the need for further research into systemic therapies to improve outcomes in this patient population.

## Introduction

Melanoma involving the gastrointestinal (GI) tract is an exceedingly rare and clinically challenging entity, often presenting with nonspecific symptoms that can delay diagnosis and treatment. The nonspecific clinical presentation of GI melanoma often leads to delayed diagnosis [1,2]. Common symptoms include chronic abdominal pain, GI bleeding (melena, hematochezia, or occult blood), weight loss, and anemia [3,4]. Acute presentations, such as intestinal obstruction, intussusception, or perforation, are rare but critical to recognize, as they often necessitate urgent surgical intervention [5]. The lack of standardized diagnostic criteria and the absence of a definitive systemic therapy further complicate the management of this aggressive disease [6].

Primary GI melanomas are even rarer, with some authors questioning their existence due to the difficulty in distinguishing them from metastatic lesions [7,8]. Diagnostic criteria for primary GI melanoma include the absence of concurrent cutaneous or extraintestinal melanoma, solitary lesions, and intramucosal involvement. However, these criteria are rarely met, and most cases are diagnosed as metastatic disease [9]. This case report addresses the diagnostic and therapeutic challenges of GI melanoma through the presentation of a 74-year-old male with metastatic melanoma of unknown primary origin, who presented with acute intestinal obstruction. The discussion contextualizes this case within the existing literature, highlighting the importance of early recognition, surgical management, and the need for advancements in systemic therapies.

## Case presentation

A 74-year-old male with a past medical history of systemic arterial hypertension, radical prostatectomy, and cholecystectomy with hernioplasty presented with right inguinal lymphadenopathy in 2021. An excisional biopsy initially suggested poorly differentiated carcinoma. However, the diagnosis required interpretation by three pathologists, ultimately identifying melanoma with chondrosarcomatous differentiation. The patient was treated with a regimen of ipilimumab and nivolumab, achieving partial response maintained for two years.

In October 2023, the patient was admitted for intermittent subocclusive symptoms. A CT scan showed no evidence of mechanical obstruction; endoscopy and colonoscopy revealed immunotherapy-associated enteritis and colitis, as well as benign colonic polyps. Symptoms resolved with low-dose prednisone, and the nivolumab regimen was continued.

**Figure 1:**
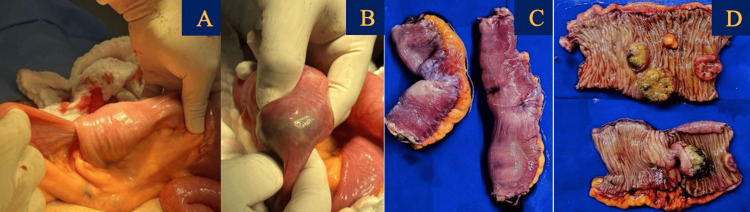
(A, B) Resection of the intestinal tumor during laparotomy. (C) Intestinal segment of 15 cm length x 4 cm maximum thickness, and segment of intestine 12 cm long x 5 cm of maximum thickness. (D) Top image: five tumor nodulations of variable dimensions of intramural location ranging from 1 x 0.8 x 0.7 cm to 3.5 x 3.2 x 2.5 cm on average; bottom image: a pedunculated tumor lesion with hyperpigmentation and necrosis which measures 4 x 3 x 2 x 2.5 cm in major axis and a wall thickness of 5 mm.

**Figure 2:**
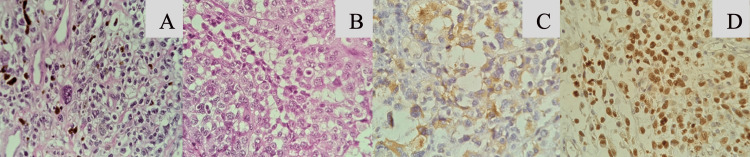
Microphotographs (A) and (B) are histologic sections stained with hematoxylin and eosin showing metastatic infiltration by histologically malignant high-grade neoplasm. Small bowel wall with multiple metastatic infiltration by high-grade malignant neoplasm immunoreactive for HMB45 (C) and SOX10 (D), with 99-100% specificity for melanoma.

In January 2024, the patient was hospitalized following syncope, pallor, hypotension, melena, and a hemoglobin level of 5.8 g/dL. Abdominal CT identified small bowel intussusception, which required an urgent open laparotomy with omental resection (Figure 1). Biopsy samples were sent for histopathological analysis, confirming malignant melanoma positive for SOX10, HMB45, and KI67 markers (Figure 2).

## Discussion

This case highlights the diagnostic and therapeutic challenges of GI melanoma, a rare and aggressive malignancy with a poor prognosis. While melanomas are most commonly associated with cutaneous origins, they can metastasize to the GI tract, accounting for 1-3% of all malignant neoplasms of the digestive system [1]. Autopsy studies reveal that 50-60% of patients with advanced melanoma have GI involvement, yet clinical diagnosis ante mortem is made in only 1-5% of cases, highlighting the rarity and diagnostic difficulty of this condition [2], often originating from an unknown primary site, as seen in this patient. The nonspecific clinical presentation, including chronic abdominal pain, GI bleeding, and acute obstruction, underscores the importance of maintaining a high index of suspicion in patients with a history of melanoma [1-4].

The diagnostic criteria for primary GI melanoma, as proposed by Blecker et al., include the absence of concurrent cutaneous or extraintestinal melanoma, solitary lesions, and intramucosal involvement. However, these criteria are rarely met, and most cases are diagnosed as metastatic disease [8,9]. In this case, the absence of a primary lesion and the presence of multiple intestinal nodules supported the diagnosis of metastatic melanoma. The immunohistochemical findings, including positivity for SOX10, HMB45, and Ki67, further confirmed the diagnosis [3].

Surgical resection remains the cornerstone of treatment for both primary and metastatic GI melanoma, particularly in cases of obstruction or bleeding. Wide resection with adequate margins and lymphadenectomy is recommended to achieve local control. However, the role of systemic therapies, including immunotherapy and targeted therapy, remains unclear. While these modalities have shown promise in cutaneous melanoma, their efficacy in secondary GI melanoma is limited by the disease’s aggressive biology and poor response rates [10,11].

This case aligns with prior research emphasizing the poor prognosis of GI melanoma, particularly in the setting of visceral metastases. The median survival for patients with metastatic GI melanoma is less than one year, highlighting the need for improved therapeutic strategies. Future research should focus on the molecular characterization of GI melanoma and the development of targeted therapies to improve outcomes [5-7].

## Conclusions

This case underscores that in patients with a history of melanoma, even subtle or nonspecific GI symptoms warrant thorough evaluation for potential metastatic involvement. In this instance, the development of acute intestinal obstruction was the first indicator of small bowel metastases, confirmed by immunohistochemical positivity for SOX10, HMB45, and Ki67. These markers are indispensable for establishing a diagnosis when no primary cutaneous lesion is identified.

Despite temporary systemic control with immunotherapy, the emergence of GI complications illustrates the aggressive nature of melanoma metastases. Prompt surgical resection is essential for managing life-threatening complications, although the overall prognosis remains poor, often with a median survival of less than one year. This case highlights the critical need for a multidisciplinary approach and intensifies the call for further research into the molecular mechanisms driving GI dissemination and the development of more effective systemic therapies.
